# Supplementary material for: Polyketide synthase gene clusters in the ericoid mycorrhizal fungus Oidiodendron maius: genome-wide prediction, expression profiling, and functional characterization of knockout mutants
Source: Front Plant Sci. 2026 Sep 16;17:1924430. doi: 10.3389/fpls.2026.1924430 (PMC13623903; doi:10.3389/fpls.2026.1924430)
Supplement: Supplementary file 1 [file SupplementaryFile1.zip › Supplementary Materials/Supplementary Folder 1.zip!/Oidma1_AssemblyScaffolds_Repeatmasked/knownclusterblast/cluster51/jgi.p_Oidma1_137829_mibig_hits.html]

| MIBiG Protein | Description | MIBiG Cluster | MiBiG Product | % ID | % Coverage | BLAST Score | E-value |
| --- | --- | --- | --- | --- | --- | --- | --- |
| ADI24931.1 | VrtF | BGC0000168 | Viridicatumtoxin\_biosynthetic\_gene\_cluster | 53 | 100.0 | 264 | 1.4e-70 |
| AEN83886.1 | AdaD | BGC0000156 | TAN-1612\_biosynthetic\_gene\_cluster | 52 | 95.6 | 264 | 1.4e-70 |
| EAU29809.1 | predicted\_protein | BGC0001400 | Citreoviridin\_biosynthetic\_gene\_cluster | 36 | 85.6 | 146 | 5.4e-35 |
